# Relationship Between Decayed Teeth and Metabolic Syndrome: Data From 4716 Middle-Aged Male Japanese Employees

**DOI:** 10.2188/jea.JE20140132

**Published:** 2015-03-05

**Authors:** Miki Ojima, Atsuo Amano, Shu Kurata

**Affiliations:** 1 Department of Preventive Dentistry, Osaka University Graduate School of Dentistry, Suita, Osaka, Japan; 1 大阪大学大学院歯学研究科予防歯科学教室; 2 Dental Clinic, Osaka Health Support Center, Sumitomo Mitsui Banking Corporation, Osaka, Japan; 2 三井住友銀行大阪健康サポートセンター歯科診療所

**Keywords:** dental caries, dyslipidemia, hyperglycemia, hypertension, obesity

## Abstract

**Background:**

Epidemiological findings regarding the relationship between decayed teeth (DT) and metabolic syndrome (MetS) are scarce. We evaluated the relationship of DT with MetS, obesity, and MetS components in early middle-aged male Japanese employees.

**Methods:**

We cross-sectionally analyzed dental and medical health checkup results from a total of 4716 participants aged 42 or 46 years. Logistic regression models were employed to calculate the odds ratios (ORs) and 95% confidence intervals (CIs) after adjustment for age, breakfast consumption frequency, drinking habits, smoking status, and physical activity.

**Results:**

Significant differences in the prevalence of MetS, obesity determined by body mass index, and the components of MetS between participating men with and without DT were detected (all *P* < 0.01). The adjusted OR of MetS was 1.41 (95% CI, 1.14–1.74) for those with 1 or 2 DT, and 1.66 (95% CI, 1.28–2.16) for those with ≥3 DT (*P* for trend = 0.01), and this significant relationship was observed even in those without periodontal pocket formation (*P* for trend = 0.03) or missing teeth (*P* for trend = 0.02). DT was significantly related to overweight/obesity and the MetS components of hypertension, dyslipidemia, and hyperglycemia, with adjusted ORs of 1.35 (95% CI, 1.19–1.53), 1.22 (95% CI, 1.07–1.39), 1.18 (95% CI, 1.03–1.34), and 1.33 (95% CI, 1.13–1.56), respectively. In addition, even in non-overweight/non-obese men, DT was found to be related to dyslipidemia and hyperglycemia, though with marginal significance (*P* < 0.05).

**Conclusions:**

Our findings suggest that having DT is related to MetS in early middle-aged Japanese men directly and through obesity and is independent of health behaviors, periodontal condition, and tooth loss.

## Abstract (ja)

**【背景】:**

未処置齲蝕とメタボリックシンドロームとの関連についての疫学報告は少ない。本研究では、中年早期の日本人男性労働者における未処置齲蝕とメタボリックシンドローム、肥満およびメタボリックシンドロームの構成因子との関連性を調べた。

**【方法】:**

歯科と医科健診を受けた42歳と46歳の4716人の健診結果を断面的に分析した。オッズ比と95％信頼区間を、ロジスティック回帰モデルを用いて年齢、朝食頻度・飲酒・喫煙・運動習慣で調整して算出した。

**【結果】:**

メタボリックシンドローム、肥満（BMIによる評価）およびメタボリックシンドロームの構成因子の有病率について、未処置齲蝕を持つ者と持たない者の間に有意な差が認められた(全てP<0.01)。メタボリックシンドロームの調整オッズ比は、1-2本の未処置齲蝕を持つ者で1.41（95%信頼区間1.14-1.74）、3本以上の未処置齲蝕を持つ者で1.66（95%信頼区間1.28-2.16）を示した(P for trend=0.01)。この関連性は歯周ポケットを持たない者（P for trend=0.03）や歯の喪失がない者においても認められた（P for trend=0.02）。未処置齲蝕は、過体重／肥満とメタボリックシンドロームの構成因子である高血圧、脂質異常、高血糖とも有意に関連し、その調整オッズ比はそれぞれ1.35 (95%信頼区間, 1.19-1.53), 1.22 (95%信頼区間, 1.07-1.39), 1.18 (95%信頼区間, 1.03-1.34), and 1.33 (95%信頼区間, 1.13-1.56)であった。また、非肥満者においても、未処置齲蝕と脂質異常・高血糖との間に、僅かではあるが有意な関連が認められた(P<0.05)。

**【結論】:**

中年早期の日本人男性において、未処置齲蝕は直接あるいは肥満を介してメタボリックシンドロームと関連し、その関連は健康習慣や歯周状態・歯の喪失とは独立したものであることが示唆された。

## INTRODUCTION

Metabolic syndrome (MetS) is an established risk factor for atherosclerosis, which greatly contributes to cardiovascular disease. Between 20% and 30% of the adult population has MetS in most countries worldwide,^1^ and the high prevalence of MetS is a global public health problem. Since atherosclerosis typically occurs in middle age or later, prevention and control of MetS in early middle-aged individuals is important to reduce the health burden of cardiovascular disease.

Over the past two decades, increasing epidemiological evidence linking poor oral health and systemic conditions has been presented, with a large amount of research focusing on the possible relationships of periodontal disease with cardiovascular disease^2^ and diabetes.^3^ Periodontal disease is also related to various atherosclerotic risk factors, including obesity, hypertension, dyslipidemia, hyperglycemia, and MetS.^4^ One proposed biological pathway linking periodontal disease and atherosclerosis is chronic inflammatory response caused by periodontal pathogens such as *Porphyromonas gingivalis*.^5^

Dental caries is the most commonly encountered oral disease, and 25%–50% of middle-aged adults in developed countries have decayed teeth (DT).^6^^–^^8^ Recently, *Streptococcus mutans*, a cariogenic dental pathogen, was detected in human atherosclerotic lesions.^9^^,^^10^ Furthermore, infection with an invasive strain of *S. mutans* was shown to accelerate atherosclerotic plaque development and inflammatory cell invasion in a mouse model.^11^ Although it can be inferred from these studies that individuals with DT show a greater risk of atherosclerogenesis than those without DT, epidemiological evidence supporting this notion is very scarce.^12^^,^^13^ We therefore obtained data from health checkups of 42- and 46-year-old male employees and evaluated the relationship of DT with MetS, obesity, and MetS components.

## METHODS

### Data source and study population

The present cross-sectional study was performed using records obtained from health checkups conducted at a large financial company in Japan. In Japan, the Industrial Safety and Health Law requires employers to provide annual medical checkups to employees. Although dental checkups are not required by the law, employees of the company had two dental checkups in their 40s, at the ages of 42 and 46 years. The study population consisted of all male workers aged 42 and 46 years who participated in the health checkup from April 2008 to December 2011 (*n* = 5045). Of the 5045 participants, 329 were excluded from the analyses due to lack of data from the dental examination. Therefore, we analyzed data from 4716 eligible participants who underwent both medical and dental checkups.

This study was approved by the Ethics Committee of Osaka University, Graduate School of Dentistry (H24-E18). Employees of the company gave consent for their health records to be stored and used for future research, and our Ethics Committee and the Department of Health and Welfare of the company approved this retrospective review of the health checkup records of all employees whose data were included in this study. The need for specific written informed consent from the participants was waived by the ethics committee and was not obtained, as the dataset consisted of de-identified secondary data for research purposes. Instead, we published all relevant information regarding the conduct of the study and provided each prospective participant an opportunity to refuse inclusion in the study. That publication was made available for 12 months prior to the study, so that the prospective participants could readily obtain the information.

### Medical checkups

The health checkups were conducted at the health support centers of the company branches in Osaka and Tokyo. Each medical checkup included a self-administered questionnaire regarding health behaviors, a physical examination, and collection of blood samples for laboratory analysis. The self-administered questionnaire consisted of four parts: drinking habit, smoking status, physical activity, and dietary pattern. The physical examination included measurements of blood pressure (BP), height, body weight, and waist circumference (WC). Nurses measured the BP in each participant in a sitting position after resting for at least 5 minutes using an automatic electronic BP monitor (HM-701; Elquest Corporation, Chiba, Japan), which was calibrated every week. A minimum of two measurements were taken on outstretched arms at 1–2 minute intervals. If the readings were high (systolic BP ≥130 mm Hg or diastolic BP ≥85 mm Hg), then additional readings were obtained. The lowest readings were recorded. WC was measured to the nearest 0.1 cm using an inelastic tape at the level of the umbilicus with the participant standing and breathing normally. Standard enzymatic methods were used to determine the serum concentrations of high-density lipoprotein cholesterol (HDL-C), triglycerides (TG), and fasting blood glucose (FBG). The concentrations of HDL-C and TG were measured with commercial kits (Sekisui Medical Co., Ltd., Tokyo, Japan) and an automated analyzer (7180; Hitachi High-Technologies Corporation, Tokyo, Japan). FBG concentration was measured using an enzymatic method (JCA-BM6010; JEOL Ltd., Tokyo, Japan).

### Dental checkups

Each participant was asked about dental health behaviors, including frequency of daily brushing, and underwent a dental examination at the health support center dental clinic by one of eight dentists appropriately trained according to standardized procedures recommended in the manual published by the World Health Organization.^14^ Dental caries were diagnosed with a plain mouth mirror and a community periodontal index (CPI) probe. Inter-examiner reliability for assessments of dental caries showed that Cohen κ values ranged from 0.96 to 1.00. DT for all teeth were recorded when there was a lesion in a pit or fissure on a smooth tooth surface, an unmistakable cavity, undermined enamel, a detectably softened floor or wall, or a soft or leathery feel upon CPI probing. Missing teeth were defined as permanent teeth lost due to extraction or dropout. Although assessment of periodontal condition was not mandatory as part of the dental checkup, CPI was determined for 82% (*n* = 4606) of the participants. The periodontal condition of 10 index teeth was assessed by CPI, with CPI codes of 0–4 used (0, healthy gingiva; 1, gingival bleeding on probing; 2, calculus; 3, periodontal pocket 4–5 mm; 4, periodontal pocket ≥6 mm).^15^ The highest CPI code value among the 6 sections served as the score for the participant.

### Potential confounders

Dental caries and MetS may share common risk factors, including poor health behaviors such as increased alcohol consumption, smoking, low physical activity, and poor dietary habits.^16^ Based on a self-administered questionnaire, participants were classified as non- or current drinker, while smoking status was divided into never, former, and current. Physical activity was classified as ≥1 or <1 time/week based on frequency of exercise. Breakfast consumption frequency was used as a marker of poor dietary habits^17^ and divided into two categories: every day or never/sometimes.

### Data analyses

Based on the dental examination findings, the participants were divided into two groups: those with and those without DT. Third molars were excluded from the analysis. Body mass index (BMI) was calculated by dividing weight (in kilograms) by height (in meters) squared. MetS was defined according to the criteria of the American Heart Association Scientific Statements of 2009.^18^ The participants were considered to have MetS if they presented ≥3 of the following components: (1) central obesity (≥90.0 cm, the WC cut point for Asian populations recommended by the International Diabetes Federation), (2) elevated TG (≥150 mg/dL), (3) reduced HDL-C (<40 mg/dL), (4) elevated BP (systolic BP ≥130 mm Hg and/or diastolic BP ≥85 mm Hg), and (5) elevated FBG (≥100 mg/dL). Participants receiving drug treatment for a component were regarded as having the component.

We defined MetS as a primary outcome and used secondary outcomes to confirm and support the result based on the primary outcome. The secondary outcomes included overweight/obesity (BMI ≥25.0 kg/m^2^), central obesity, hypertension (elevated BP), dyslipidemia (elevated TG and/or reduced HDL-C), and hyperglycemia (elevated FBG). The characteristics of the participants were compared between those with and without DT using a chi-square test for categorical variables and a *t*-test for continuous variables. Logistic regression models were constructed to calculate the odds ratios (ORs) and 95% confidence intervals (CIs) for MetS, overweight/obesity, central obesity, hypertension, dyslipidemia, and hyperglycemia. The models included age, breakfast consumption frequency, drinking habit, smoking status, and physical activity as adjusted variables. Dependent variables were defined in a binary fashion: participants without the outcome were given a score of 0, and those with the outcome were given a score of 1. In the model for MetS, the independent variable of DT was divided into three categories based on the number of DT: 0, 1 or 2, and ≥3. Since a relationship between frequency of toothbrushing and MetS has been reported,^19^ frequency of toothbrushing (<3 and ≥3 times/day) as an adjusted variable was included in the model. Two additional analyses among only participants without periodontal pocket formation (CPI of <3) and those without missing teeth were carried out to control for the confounding effects of periodontal disease and tooth loss, respectively. For hypertension, dyslipidemia, and hyperglycemia, additional models were constructed, for non-overweight/obese and non-centrally obese participants, to control for the confounding effect of obesity. Interactions between DT and potential confounders were tested by adding cross-product terms to each model. Complete data on all variables were used in all analyses. The goodness of fit for multivariate models was verified by the Hosmer-Lemshow statistic. Statistical analyses were performed using the PASW Statistics 18 statistical software package (IBM Corp., New York, NY, USA), with the significance level set at *P* < 0.05 for a two-sided test. The study conforms to STROBE guidelines for cross-sectional studies.^20^

## RESULTS

Of the 4716 participants, there were 2886 (61.2%) without and 1830 (38.8%) with DT. The mean number of DT among those with DT was 2.51 (standard deviation, 2.47). Table 1 compares the characteristics of participants according to the presence of DT. There were fewer healthy behaviors (except for drinking habit), lower frequency of brushing per day, a higher prevalence of missing teeth, and poorer periodontal condition (CPI ≥3) (all *P* < 0.01) in participants with DT than in those without. Furthermore, higher values for BMI, WC, SBP, DBP, TG, and FBS, and lower levels of HDL-C were observed in participants with DT than in those without (all *P* < 0.05). Also, participants with DT more frequently received drug treatment for hyperglycemia than those without.

**Table 1.** Characteristics of study population according to the presence of decayed teeth

Characteristics	Without DT	With DT	*P* value^a^
Number of participants	2886	1830	
Age			
42 years old	1277 (59.6)	867 (40.4)	0.04
46 years old	1609 (62.6)	963 (37.4)	
Breakfast consumption frequency (*n*, %)			
Everyday	2308 (62.9)	1364 (37.1)	<0.01
Never or sometimes	577 (55.3)	466 (44.7)	
Drinking habit (*n*, %)			
None	439 (60.5)	287 (39.5)	0.67
Current	2446 (61.3)	1543 (38.7)	
Smoking status (*n*, %)			
Never	1436 (65.4)	759 (34.6)	<0.01
Former	695 (62.0)	426 (38.0)	
Current	753 (53.9)	645 (46.1)	
Physical activity (*n*, %)			
≥1 time/week	1470 (64.3)	815 (35.7)	<0.01
<1 time/week	1415 (58.3)	1012 (41.7)	
Frequencies of daily brushing (*n*, %)			
≥3 times/day	992 (65.9)	513 (34.1)	<0.01
<3 times/day	1833 (58.4)	1307 (41.6)	
Number of missing teeth (*n*, %)			
0	2233 (63.3)	1297 (36.7)	<0.01
≥1	653 (55.1)	533 (44.9)	
Community Periodontal Index^b^ (*n*, %)			
0, 1, or 2	1862 (61.8)	1149 (38.2)	<0.01
3 or 4	528 (56.5)	407 (43.5)	
Body mass index (kg/m^2^) (mean, SD^c^)	23.7 (3.1)	24.2 (3.3)	<0.01
Waist circumference (cm) (mean, SD)	84.1 (8.2)	85.4 (8.8)	<0.01
Systolic blood pressure (mm Hg) (mean, SD)	120.8 (13.8)	121.9 (14.9)	0.01
Diastolic blood pressure (mm Hg) (mean, SD)	75.8 (10.2)	76.6 (11.0)	0.02
HDL cholesterol (mg/dL) (mean, SD)	57.1 (13.4)	55.4 (13.0)	<0.01
Triglycerides (mg/dL) (mean, SD)	111.8 (85.4)	123.4 (99.1)	<0.01
Fasting blood glucose (mg/dL) (mean, SD)	91.9 (14.7)	93.6 (16.9)	<0.01
Under drug treatment for hypertension (*n*, %)	220 (58.7)	155 (41.3)	0.30
Under drug treatment for dyslipidemia (*n*, %)	265 (59.4)	181 (40.6)	0.42
Under drug treatment for hyperglycemia (*n*, %)	67 (51.1)	64 (48.9)	0.02

DT, decayed teeth; HDL, high-density lipoprotein; SD, standard deviation.

^a^Chi-square test for categorical variables and *t*-test for continuous variables.

^b^Number of participants in whom the index was measured: 3946.

^c^Standard deviation.

Number of missing data: breakfast consumption frequency (1), drinking habit (1), smoking status (2), physical activity (4), frequencies of daily brushing (71).

Table 2 shows the prevalence of MetS, obesity (determined by BMI and WC), and the components of MetS according to the presence of DT. Participants with DT showed a higher prevalence of MetS, overweight/obesity, central obesity, hypertension, dyslipidemia, and hyperglycemia than those without DT (all *P* < 0.01).

**Table 2.** Prevalence of metabolic syndrome, obesity, and its components according to the presence of decayed teeth

		Proportion (%)	*P* value^a^
MetS^b^	Total	521/4716 (11.0)	
	Without DT	264/2886 (9.1)	<0.01
	With DT	257/1830 (14.0)	

Overweight/obesity^c^	Total	1487/4716 (31.5)	
	Without DT	830/2886 (28.8)	<0.01
	With DT	657/1830 (35.9)	

Central obesity^d^	Total	1139/4716 (24.2)	
	Without DT	622/2886 (21.6)	<0.01
	With DT	517/1830 (28.3)	

Hypertension^e^	Total	1400/4716 (29.7)	
	Without DT	815/2886 (28.2)	<0.01
	With DT	585/1830 (32.0)	

Dyslipidemia^f^	Total	1319/4716 (28.0)	
	Without DT	760/2886 (26.3)	<0.01
	With DT	559/1830 (30.5)	

Hyperglycemia^g^	Total	737/4716 (15.6)	
	Without DT	406/2886 (14.1)	<0.01
	With DT	331/1830 (18.1)	

DT, decayed teeth; MetS, metabolic syndrome.

^a^Chi-square test.

^b^Presence ≥3 of the following components: (1) central obesity, (2) elevated triglycerides, (3) reduced HDL cholesterol, (4) elevated blood pressure [systolic blood pressure ≥130 mm Hg and/or diastolic blood pressure ≥85 mm Hg], and (5) elevated fasting blood glucose.

^c^Body mass index ≥25.0 kg/m^2^.

^d^Waist circumference ≥90.0 cm.

^e^Systolic blood pressure ≥130 mm Hg and/or diastolic blood pressure ≥85 mm Hg, or treated for hypertension.

^f^Triglycerides ≥150 mg/dl and/or HDL cholesterol <40 mm Hg, or treated for dyslipidemia.

^g^Fasting blood glucose ≥100 mg/dl or treated for hyperglycemia.

Table 3 shows crude and adjusted ORs for MetS according to number of DT. Among all participants, the number of DT was significantly related to increased risk of MetS. The adjusted OR of MetS was 1.41 (95% CI, 1.14–1.74) for those with 1 or 2 DT, and 1.66 (95% CI, 1.28–2.16) for those with ≥3 DT (*P* for trend = 0.01). When the analysis was limited to participants without periodontal pocket formation, a relationship between DT and MetS was also observed. The adjusted OR of MetS was 1.54 (95% CI, 1.18–1.99) for those with 1 or 2 DT, and 1.69 (95% CI, 1.19–2.41) for those with ≥3 DT (*P* for trend = 0.03). Similarly, in participants without missing teeth, a relationship between DT and MetS was found with an adjusted OR of 1.41 (95% CI, 1.10–1.81) for those with 1 or 2 DT and 1.75 (95% CI, 1.27–2.40) for those with ≥3 DT (*P* for trend = 0.02).

**Table 3.** Odds ratio for metabolic syndrome^a^ according to number of decayed teeth

	Crude OR (95% CI)	Adjusted^b^ OR (95% CI)
All participants (*n* = 4640)		
Number of DT		
0 (*n* = 2823)	1.00 (Reference)	1.00 (Reference)
1 or 2 (*n* = 1245)	1.51 (1.23–1.86)	1.41 (1.14–1.74)
≥3 (*n* = 572)	1.88 (1.45–2.42)	1.66 (1.28–2.16)
*P* for trend	<0.01	0.01

Participants without periodontal pocket formation^c^ (*n* = 2993)		
Number of DT		
0 (*n* = 1851)	1.00 (Reference)	1.00 (Reference)
1 or 2 (*n* = 818)	1.60 (1.23–2.07)	1.54 (1.18–1.99)
≥3 (*n* = 324)	1.86 (1.32–2.63)	1.69 (1.19–2.41)
*P* for trend	<0.01	0.03

Participants without missing teeth (*n* = 3467)		
Number of DT		
0 (*n* = 2176)	1.00 (Reference)	1.00 (Reference)
1 or 2 (*n* = 906)	1.50 (1.17–1.91)	1.41 (1.10–1.81)
≥3 (*n* = 385)	1.96 (1.43–2.67)	1.75 (1.27–2.40)
*P* for trend	<0.01	0.02

CI, confidence interval; DT, decayed teeth; OR, odds ratio.

^a^Presence ≥3 of the following components: (1) central obesity, (2) elevated triglycerides, (3) reduced HDL cholesterol, (4) elevated blood pressure [systolic blood pressure ≥130 mm Hg and/or diastolic blood pressure ≥85 mm Hg], and (5) elevated fasting blood glucose.

^b^Adjusted for age (42 or 46 years old), breakfast consumption frequency (everyday or never/sometimes), drinking habit (none or current), smoking status (never, former or current), physical activity (≥1 time/week or <1 time/week), and frequency of tooth-brushing (≥3 times/day or <3 times/day).

^c^Community Periodontal Index <3.

Table 4 shows crude and adjusted ORs for overweight/obesity and central obesity according to the presence of DT. The adjusted OR was 1.35 (95% CI, 1.19–1.53) for overweight/obesity and 1.38 (95% CI, 1.21–1.58) for central obesity. The main effect of DT on central obesity was qualified by a significant interaction between DT and physical activity.

**Table 4.** Odds ratio for obesity according to the presence of decayed teeth

Obesity	Overweight/obesity^a^	Central obesity^b^
Crude OR (95% CI)		
Without DT	1.00 (Reference)	1.00 (Reference)
With DT	1.39 (1.22–1.57)	1.43 (1.25–1.64)
*P* value	<0.01	<0.01
Adjusted^c^ OR (95% CI)		
Without DT	1.00 (Reference)	1.00 (Reference)
With DT	1.35 (1.19–1.53)	1.38 (1.21–1.58)^d^
*P* value	<0.01	<0.01

CI, confidence interval; DT, decayed teeth; OR, odds ratio.

^a^Body mass index ≥25.0 kg/m^2^.

^b^Waist circumference ≥90.0 cm.

^c^Adjusted for age (42 or 46 years old), breakfast consumption frequency (everyday or never/sometimes), drinking habits (none or current), smoking status (never, former or current), and physical activity (≥1 time/week or <1 time/week).

^d^Qualified by a significant interaction between DT and physical activity.

Crude and adjusted ORs for MetS components among participants with and without DT are shown in Table 5. Among all participants, those with DT showed significantly higher prevalence and adjusted ORs in all MetS components than those without DT (*P* < 0.05). When the analysis was limited to non-overweight/obese men, marginally significant relationships between DT and dyslipidemia and hyperglycemia were found with adjusted ORs of 1.20 (95% CI, 1.00–1.43) and 1.28 (95% CI, 1.01–1.62), respectively. In participants who did not exhibit central obesity, those with DT had higher adjusted ORs for dyslipidemia and hyperglycemia than those without DT, but the difference was not significant.

**Table 5.** Odds ratio for metabolic syndrome components according to the presence of decayed teeth

MetS components	Hypertension^a^	Dyslipidemia^b^	Hyperglycemia^c^
All participants (*n* = 4711)			
Crude OR (95% CI)			
Without DT	1.00 (Reference)	1.00 (Reference)	1.00 (Reference)
With DT	1.19 (1.05–1.36)	1.23 (1.08–1.40)	1.35 (1.15–1.58)
*P* value	<0.01	<0.01	<0.01
Adjusted^d^ OR (95% CI)			
Without DT	1.00 (Reference)	1.00 (Reference)	1.00 (Reference)
With DT	1.22 (1.07–1.39)	1.18 (1.03–1.34)	1.33 (1.13–1.56)
*P* value	<0.01	0.02	<0.01

Non-overweight/obese^e^ participants (*n* = 3226)			
Crude OR (95% CI)			
Without DT	1.00 (Reference)	1.00 (Reference)	1.00 (Reference)
With DT	1.05 (0.88–1.25)	1.24 (1.05–1.48)	1.20 (1.00–1.58)
*P* value	0.57	0.01	0.05
Adjusted OR (95% CI)			
Without DT	1.00 (Reference)	1.00 (Reference)	1.00 (Reference)
With DT	1.10 (0.92–1.31)	1.20 (1.00–1.43)	1.28 (1.01–1.62)
*P* value	0.32	<0.05	<0.05

Non-centrally obese^f^ participants (*n* = 3573)			
Crude OR (95% CI)			
Without DT	1.00 (Reference)	1.00 (Reference)	1.00 (Reference)
With DT	1.02 (0.87–1.20)	1.20 (1.02–1.41)	1.21 (0.98–1.49)
*P* value	0.79	0.03	0.08
Adjusted OR (95% CI)			
Without DT	1.00 (Reference)	1.00 (Reference)	1.00 (Reference)
With DT	1.05 (0.89–1.24)	1.15 (0.98–1.34)	1.22 (0.99–1.51)
*P* value	0.54	0.09	0.07

CI, confidence interval; DT, decayed teeth; MetS, metabolic syndrome; OR, odds ratio.

^a^Systolic blood pressure ≥130 mm Hg and/or diastolic blood pressure ≥85 mm Hg, or treated for hypertension.

^b^Triglycerides ≥150 mg/dl and/or HDL cholesterol <40 mm Hg, or treated for dyslipidemia.

^c^Fasting blood glucose ≥100 mg/dl or treated for hyperglycemia.

^d^Adjusted for age (42 or 46 years old), breakfast consumption frequency (everyday or never/sometimes), drinking habits (none or current), smoking status (never, former or current), and physical activity (≥1 time/week or <1 time/week).

^e^Body mass index <25.0 kg/m^2^.

^f^Waist circumference <90.0 cm.

## DISCUSSION

The present analysis of health checkup results of middle-aged Japanese male employees showed that the number of DT was significantly related to increased odds of MetS after adjustments for health behaviors as potential confounders, and this connection was observed even in participants without periodontal pocket formation and those without missing teeth. These results indicate that DT is related to MetS in men, independent of health behaviors, periodontal condition, and tooth loss. In addition, DT was significantly related to overweight/obesity and the MetS components hypertension, dyslipidemia, and hyperglycemia. To the best of our knowledge, only one study has previously investigated the relationship between dental caries and MetS and its components in adults.^12^ In contrast to our results, the previous study of Finnish adults aged 30 to 64 years did not find any significant relationship between dental caries and MetS and its components after adjustment for potential confounders, although MetS, insulin resistance, central obesity, hypertension, and hyperglycemia were positively and weakly related with dental caries. This may be due in part by sample and methodological differences from our study, including enrollment only of non-diabetic participants who had never smoked and data analysis of men and women together.

Dental caries is a major cause of tooth loss. Furthermore, several studies have reported that number of teeth is inversely associated with cardiometabolic risks^21^ and cardiovascular disease-related mortality.^22^^,^^23^ One explanation for this association may be because tooth loss is a surrogate marker for periodontal disease, since tooth loss due to periodontal disease is predominant in adults after reaching middle age. However, a nationwide study in Japan demonstrated that around 30% of teeth were extracted due to dental caries even in groups over 45 years of age.^24^ Our finding suggests that the presence of dental caries also contributes to the association of tooth loss with cardiovascular disease.

In the present study, DT was shown to be significantly related to overweight/obesity. This finding suggests that DT is related to MetS through obesity because obesity is a well-established risk factor for MetS.^25^ Although there has been extensive investigation of the relationships between dental caries and obesity in children and adolescents, very few published studies have noted this relationship in adults.^26^ A recent National Health and Nutrition Examination Survey (NHANES) report showed that adults aged ≥20 years old with unmet treatment needs for dental caries had higher odds of BMI ≥30.0 kg/m^2^ than those without.^27^ The relationship between DT and obesity has been also discussed in regard to reduction of salivary flow.^28^ Obesity is associated with salivary gland hypofunction,^29^ and hyposalivation increases susceptibility to dental caries.^30^ Additionally, DT likely causes faster eating due to oral impact, such as mastication pain and chewing difficulty,^31^ which leads to obesity^32^; however, direct evidence regarding the impact of DT on rate of eating is required to confirm this possibility.

When our analysis was limited to non-overweight/obese or non-centrally obese participants, the significance of the relationship between DT and MetS components diminished or disappeared. These results also suggest that the relation of DT on MetS components is a combined effect of DT itself and DT-related obesity, though the biological mechanism(s) involved have not been revealed. In non-overweight/obese participants, weak relationships among DT, dyslipidemia, and hyperglycemia were found with marginal significance, indicating that several possible pathways linking DT and MetS components other than obesity may exist. First, cariogenic pathogens may contribute to inflammation in vascular wall cells. A previous study showed that *S. mutans* Xc induced the production of interleukin-6 (IL-6), a key cytokine in atherosclerosis, from invaded and infected human aortic endothelial cells.^33^ IL-6 is positively associated with C-reactive protein production and hyperglycemia and negatively associated with lipoprotein lipase activity and HDL-C.^25^ Second, DT may lead to reduced intake of vegetables, which induces oxidative stress associated with metabolic disorder.^34^ In a birth cohort study conducted in Northern Finland, 31-year-old adults with DT consumed vegetables less frequently than those without.^35^ However, the effects of DT on chewing deficiencies,^36^ if any, may have been limited in our study population, because the mean number of DT (2.5) was relatively low. Third, increased glucose concentration in gingival crevicular fluid may be related to increased risk for decay. Systemic blood glucose concentration has been shown to be comparable to that found in gingival crevicular blood.^37^ Finally, the oral impact of DT may produce chronic stress,^38^ which could induce MetS.^39^

There are several limitations to this study. First, the design was cross-sectional. The time sequence between DT and MetS could not be assessed because we did not have information about the period in which dental caries were left untreated, so a causal association between DT and MetS should not be assumed. Second, sugar intake, which is a common risk factor for dental caries and MetS,^40^ was not investigated in this study because the information was not included in the health check-up data. Instead, we used breakfast consumption frequency as a surrogate. An NHANES study from 1999–2002 demonstrated that the percentage of energy intake from added sugars was higher in breakfast skippers than in breakfast consumers,^17^ although the age and race of those subjects were different from those in the present study. Third, no multiplicity adjustments were performed, since one primary outcome was defined in this study.^41^ However, the possibility of type I error cannot be entirely ruled out because a number of logistic regression models were constructed. Fourth, the inherent under-estimation of periodontal pockets may occur by examining only index teeth and measuring only periodontal probing depth, not clinical attachment loss. Fifth, participants in our study were 42- and 46-year-old Japanese workers employed by a large financial company, thus our results may not be applicable to other demographic groups. Finally, socioeconomic status (SES) such as education and income was not taken into account, although SES is reported to be associated with DT^42^ as well as MetS.^43^ However, our study population is considered to have only small variations in SES, as all participants were bankers with similar ages and employed by the same company.

In conclusion, the relationship of DT with MetS was observed in early middle-aged Japanese male employees. Dental professionals are encouraged to provide education regarding the adverse effects of DT, as well as the importance of dental caries treatment for prevention and control of MetS through routine clinical and health service information sources. Nevertheless, because of limitations of the present study, further investigations should be conducted to examine whether DT is a true risk factor for MetS by employing a cohort design, a representative sample, and collection of detailed dietary information.

## ONLINE ONLY MATERIAL

Abstract in Japanese.
