# Supplementary material for: Relationship Between Decayed Teeth and Metabolic Syndrome: Data From 4716 Middle-Aged Male Japanese Employees
Source: J Epidemiol. 2015 Mar 5;25(3):204–11. doi: 10.2188/jea.JE20140132 (PMC4340997; doi:10.2188/jea.JE20140132)
Supplement: Abstract in Japanese. [file je-25-204-s001.pdf]

未処置齲蝕とメタボリックシンドロームとの関連性：中年期の日本人男性労働者 4716 人のデータより

小島美樹[1]、天野敦雄[1]、倉田　秀[2]

[1]大阪大学大学院歯学研究科予防歯科学教室
[2]三井住友銀行大阪健康サポートセンター歯科診療所

【背景】未処置齲蝕とメタボリックシンドロームとの関連についての疫学報告は少ない。本研究では、中年早期の日本人男性労働者における未処置齲蝕とメタボリックシンドローム、肥満およびメタボリックシンドロームの構成因子との関連性を調べた。
【方法】歯科と医科健診を受けた 42 歳と 46 歳の 4716 人の健診結果を断面的に分析した。オッズ比と 95%信頼区間を、ロジスティック回帰モデルを用いて年齢、朝食頻度・飲酒・喫煙・運動習慣で調整して算出した。
【結果】メタボリックシンドローム、肥満（BMI による評価）およびメタボリックシンドロームの構成因子の有病率について、未処置齲蝕を持つ者と持たない者の間に有意な差が認められた(全て P<0.01)。メタボリックシンドロームの調整オッズ比は、1-2 本の未処置齲蝕を持つ者で 1.41（95%信頼区間 1.14-1.74）、3 本以上の未処置齲蝕を持つ者で 1.66（95%信頼区間 1.28-2.16）を示した（P for trend=0.01）。この関連性は歯周ポケットを持たない者（P for trend=0.03）や歯の喪失がない者においても認められた（P for trend=0.02）。未処置齲蝕は、過体重／肥満とメタボリックシンドロームの構成因子である高血圧、脂質異常、高血糖とも有意に関連し、その調整オッズ比はそれぞれ 1.35 （95%信頼区間， 1.19-1.53）， 1.22 （95%信頼区間， 1.07-1.39）， 1.18 （95%信頼区間， 1.03-1.34）， and 1.33 （95%信頼区間， 1.13-1.56)であった。また、非肥満者においても、未処置齲蝕と脂質異常・高血糖との間に、僅かではあるが有意な関連が認められた(P<0.05)。
【結論】中年早期の日本人男性において、未処置齲蝕は直接あるいは肥満を介してメタボリックシンドロームと関連し、その関連は健康習慣や歯周状態・歯の喪失とは独立したものであることが示唆された。

キーワード：齲蝕、脂質異常、高血糖、高血圧、肥満
